# CYLD tumour supressor regulates ciligenesis

**DOI:** 10.1186/2046-2530-1-S1-P66

**Published:** 2012-11-16

**Authors:** T Eguether, BM Bonnet, EM Ermolaeva, PM Pasparakis, CG Courtois, TAM Tassin

**Affiliations:** 1 Institut Curie INSERM U759, France; 2 Cologne University, Germany; 3 INSERM, France

## 

The tumor suppressor CYLD gene encodes a deubiquitinating enzyme that removes lys-63-linked ubiquitin chains. CYLD regulates, by its catalytic domain, NF-kB, c-Jun kinase and Wnt/β-catenin signalling pathways. When mutated in its catalytic domain CYLD cause skin appendages tumors as in familial cylindromatosis. We have found CYLD as a partner of the centrosomal protein CAP350 in immunoprecipitation experiments followed by mass spectrometry. CAP350 was shown to play a role in microtubule stabilization (Hoppeler-Leber et al, 2007). Here, we show that CYLD localizes to the centrosome in cells and is enriched in purified centrosomes. To understand the functional interaction between CYLD and CAP350, we studied mice carrying deletion of part of the deubiquitinase domain and mimicking the human pathology. These homozygous mice *cyld* (*del17/del17*) die perinatally due to respiratory dysfunction and exhibit immature lung phenotype. To test if CYLD is involved in ciliogenesis, we studied the presence of motile cilia in the trachea. *Cyld* (*del17/del17*) embryo tracheas exhibit a decreased number of ciliated cells compared to wild type ones of the same litter. In addition, these ciliated cells have fewer and shorter cilia. Similar results were obtained on ependymal cell culture. Transmission electron micrographs demonstrate that most of the basal bodies fail to anchor to the plasma membrane. We demonstrate that in MEFs derived from *cyld* (*del17/del17*) embryo, CYLD and CAP350 do not longer interact in contrast to MEFs derived from wild type embryo. In addition, these mutant MEFs exhibit primary cilia growth defect compared to MEFs derived from wild type mice.

